# Descriptive study of sedentary behaviours in 35,444 French working adults: cross-sectional findings from the ACTI-Cités study

**DOI:** 10.1186/s12889-015-1711-8

**Published:** 2015-04-14

**Authors:** Madina Saidj, Mehdi Menai, Hélène Charreire, Christiane Weber, Christophe Enaux, Mette Aadahl, Emmanuelle Kesse-Guyot, Serge Hercberg, Chantal Simon, Jean-Michel Oppert

**Affiliations:** Research Centre for Prevention and Health, The Capital Region of Denmark, Glostrup, Denmark; Université Paris 13, Sorbonne Paris Cité - EREN (Equipe de Recherche en Epidémiologie Nutritionnelle), U1153 Inserm, Inra, Cnam, Centre de Recherche en Epidémiologie et Biostatistiques; CRNH IdF, Bobigny, France; Paris-Est Créteil University, Department of Geography, Lab-Urba, Urbanism Institute of Paris, Paris, France; Laboratoire Image, Ville et Environnement, Université de Strasbourg, Strasbourg, France; Department of Public Health, Faculty of Health Sciences, University of Copenhagen, Copenhagen, Denmark; Department of Public Health, Hôpital Avicenne (AP-HP), Bobigny, France; CARMEN, INSERM U1060/Université de Lyon 1/INRA U1235 Lyon, Lyon, France; Université Pierre et Marie Curie-Paris 6, Department of Nutrition Pitié-Salpêtrière Hospital (AP-HP), Centre for Research on Human Nutrition Ile-de-France (CRNH IdF), Institute of Cardiometabolism and Nutrition (ICAN), Paris, France

**Keywords:** Sedentary behaviours, Lifestyle, Occupation, Perceptions, Web-based cohort, Adult, France

## Abstract

**Background:**

Given the unfavourable health outcomes associated with sedentary behaviours, there is a need to better understand the context in which these behaviours take place to better address this public health concern. We explored self-reported sedentary behaviours by type of day (work/non-work), occupation, and perceptions towards physical activity, in a large sample of adults.

**Methods:**

We assessed sedentary behaviours cross-sectionally in 35,444 working adults (mean ± SD age: 44.5 ± 13.0 y) from the French NutriNet-Santé web-based cohort. Participants self-reported sedentary behaviours, assessed as domain-specific sitting time (work, transport, leisure) and time spent in sedentary entertainment (TV/DVD, computer and other screen-based activities, non-screen-based activities) on workdays and non-workdays, along with occupation type (ranging from mainly sitting to heavy manual work) and perceptions towards physical activity. Associations of each type of sedentary behaviour with occupation type and perceptions towards physical activity were analysed by day type in multiple linear regression analyses.

**Results:**

On workdays, adults spent a mean (SD) of 4.17 (3.07) h/day in work sitting, 1.10 (1.69) h/day in transport sitting, 2.19 (1.62) h/day in leisure-time sitting, 1.53 (1.24) h/day viewing TV/DVDs, 2.19 (2.62) h/day on other screen time, and 0.97 (1.49) on non-screen time. On non-workdays, this was 0.85 (1.53) h/day in transport sitting, 3.19 (2.05) h/day in leisure-time sitting, 2.24 (1.76) h/day viewing TV/DVDs, 1.85 (1.74) h/day on other screen time, and 1.30 (1.35) on non-screen time. Time spent in sedentary behaviours differed by occupation type, with more sedentary behaviour outside of work (both sitting and entertainment time), in those with sedentary occupations, especially on workdays. Negative perceptions towards physical activity were associated with more sedentary behaviour outside of work (both sitting and entertainment time), irrespective of day type.

**Conclusions:**

A substantial amount of waking hours was spent in different types of sedentary behaviours on workdays and non-workdays. Being sedentary at work was associated with more sedentary behaviour outside of work. Negative perceptions towards physical activity may influence the amount of time spent in sedentary behaviours. These data should help to better identify target groups in public health interventions to reduce sedentary behaviours in working adults.

## Background

Sedentary behaviour (commonly defined by both intensity (≤1.5 Metabolic Equivalent of Task) and position (sitting or reclining)) is increasingly recognized as a public health issue [1-3] of worldwide importance [4]. This may represent a global threat to public health, as sedentary behaviour has been associated with increased risk of type 2 diabetes, cardiovascular disease, some types of cancer and all-cause and cardiovascular mortality, independent of moderate to high-intensity physical activity [1,3]. However there is currently a lack of detailed information on the different types of sedentary behaviour and the context in which this takes place [5], sedentary behaviour being assessed in most previous studies by time spent sitting overall or time spent watching television [6]. Neglect of other domains, such as work and transportation, and other sedentary behaviours such as computer use, may not be negligible given the increasing time adults spend in these domains [7]. Sedentary behaviour in all domains may be important, as evidence is mounting for differential health effects of different sedentary behaviours [1,5,8-11]. In a French context, computer use was found to be favourably associated with cognitive functioning among middle-aged adults, whereas an unfavourable association was found with TV viewing [12]. It is therefore important to gain knowledge on specific sedentary behaviours separately.

The workforce is particularly exposed to long periods of sitting. Historically, adults have been active at work, while today automatization and computerization have minimized physical demand at work, at least in westernized high-income countries [13]. Some [14-16], but not all [17,18] studies indicate that sedentary workers are also more likely to have high leisure-time sitting. Along the same lines, some [19], but not all [17,18] studies indicate that physically demanding jobs are associated with more sitting during leisure-time. The relationship between occupation type and sedentary behaviour outside of work is hence unclear, and more research is needed to understand these associations. In this context, variability between workdays and non-workdays, may also influence the relationship between occupation type and sedentary behaviours, although understudied [20,21].

Besides a context-specific approach, an intrapersonal approach may also help to understand the multiple determinants operating in the different settings with which this behaviour is most prevalent [22]. It has been proposed that individuals’ perceptions, motivations and preferences towards maintaining a sedentary lifestyle may be predictive of the actual amount of sedentary behaviour [5]. Perceptions towards physical activity could contribute to understanding why some workers accumulate more time in sedentary behaviours than others [23]. Our underlying behavioural assumption is that men and women generally choose to engage in activities that they perceive as positive and that the perceptions (positive/negative) attached to physical activity could influence their choice of sedentary behaviours.

Therefore the purpose of this study was to explore and describe the prevalence of self-reported sedentary behaviours, according to type of day (work or non-workday), occupation type, and perceptions towards physical activity, in a large sample of 35,444 French working adults.

## Methods

### Ethics statement

This study was approved by the “Comité National Informatique et Liberté” (CNIL n°908450, n° 909216 and DR-2012-576). The NutriNet-Santé Study (see below) was approved by the Institutional Review Board of the French Institute for Health and Medical Research (IRB Inserm n°0000388FWA00005831). Written informed consent was obtained from all subjects.

### NutriNet-santé cohort

The NutriNet-Santé study is a large ongoing web-based prospective study launched in France in May 2009, with a scheduled follow-up of 10 years, with a main focus on studying the relationships between nutrition and chronic disease risk as well as determinants of dietary behaviours. Using a dedicated personalized website, recruitment is carried out with the aim to register up to 500,000 volunteer Internet-users, among whom 60% are expected to have complete baseline data in order to be included. Participants aged 18 years or older living in France and having access to the Internet fill in self-administered web-based questionnaires at baseline and then regularly during follow-up. A detailed description of the NutriNet-Santé cohort has been published previously [24].

### Study participants

Participants were drawn from a subgroup (n = 55,694) of the total sample of subjects from the NutriNet-Santé cohort who completed a questionnaire on physical activity and mobility, administered via Internet from February 15 to July 15 2013 (48.5% participation rate). This questionnaire was mainly designed to assess active transport and urban mobility in everyday life in the past four weeks. An automated e-mail informed participants of the necessity to complete their profiles by filling out this questionnaire (which took less than 20 min on average) in their personal space on the website of the NutriNet-Santé cohort study. Participants had previously completed baseline questionnaires on health, lifestyle and socio-demographic factors at inclusion.

The present study sample included all working participants with non-missing data on employment status, sedentary behaviours and covariates (age, sex, education level). Employment status was measured as dichotomous variable (yes/no) defined as studying or in employment (paid/unpaid) at any time during the last 4 weeks. Participants (n = 656) were excluded because of physical limitations to mobility, assessed through self-reported motor impairments (n = 407) and self-reported limitations to the ability to walk at least 100 m (n = 249). The present analyses were performed on a final sample of 35,444 working subjects (79% women) with a mean ± SD age of 44.5 ± 13.0 y.

### Sedentary behaviour

Two sedentary time exposures were investigated. *Sitting time* was assessed in the domains of *work, transport*, and *leisure*. Participants reported “hours per day usually spent sitting on an average workday/non-workday in the past four weeks: 1) during work; 2) for transport and in transit; 3) during leisure-time (TV, computer, reading, etc)”. Work sitting was only assessed for workdays. *Entertainment time* was assessed as leisure-time spent in each sedentary entertainment: *TV/DVD; other screen based*; *non-screen based*. Participants reported “hours per day usually spent (excluding working hours) on an average workday/non-workday in the past four weeks: 1) viewing television, DVDs and other videos; 2) using a computer, a tablet, and playing inactive video games; 3) sitting for reading, writing, sewing, knitting etc.”. All variables were included as continuous variables, expressed as hours per day (h/d). The validity of total sitting time (sum of the questions on work, transport and leisure sitting) has been assessed in 84 subjects against total sitting time measured by the inclinometer Actigraph GT3X + TM (ActiGraph Ltd, Pensacola, FL, US) (Spearman rho = 0.45, p < 0.0001). One-month repeatability data obtained in 32 adults showed intra-class coefficients that were moderate for TV/DVD viewing (0.73) and work sitting (0.71) but lower for other variables such as transport sitting (0.27) (data not shown).

### Occupation type

Occupation type was assessed with the question “Please choose from 1 (very sedentary work) to 5 (intense activity) what best corresponds with the intensity of physical activity demanded by your occupation in the past 4 weeks”. Response options included: 1. Mainly sitting, 2. Combination of sitting/standing, 3. Mainly standing, 4. Some physical effort, 5. Heavy manual work. This question was derived from questions on physical activity levels at work as described in questionnaires such as the EPIC-Norfolk Physical Activity Questionnaire [25] and the Recent Physical Activity Questionnaire [26].

### Perceptions towards physical activity

To examine perceptions towards physical activity we used three questions. “Do you consider yourself an active person” Yes/No; “Does the family in which you grew up attach value to physical activity and exercise?” A lot/A little/No; “Do you believe that, for a healthy lifestyle, regular physical activity is: Very important/Important/Less important?” Conceptually, these questions are measuring (1) identity or self-perception, (2) family (social) norms, and (3) the importance dimension. The items have not been assessed with standardized instruments, but are similar to the ones included in validated and commonly used SDT-based instruments (e.g., the Intrinsic Motivation Inventory [27]) which showed good internal consistency.

### Socio-demographic covariates

Socio-demographic covariates included sex, age (categorized into young adults (18-39 years), middle-aged adults (40-59 years) and older adults (≥60 years)), and education level (primary/secondary/university).

### Statistical analysis

Time spent sitting and time spent in sedentary entertainment was described as means and standard deviations (SD). Associations with sex, age, education level, occupation type and perceptions towards physical activity were tested separately for each sedentary exposure, stratified by day type (working days vs. non-working days) using multiple linear regressions. Associations with education level were adjusted for sex and age, and associations with occupation type and perception towards physical activity were adjusted for sex, age, and education level. Assumptions of normality and homogeneity of variance were examined graphically by Q-Q plot and scatterplots of residuals. Interaction with sex was tested. Only participants with complete data were included in analyses. P-values below 0.05 were considered statistically significant. Statistical analyses were performed with software package SAS (version 9.3, SAS Institute Inc., Cary, NC, USA).

## Results

Table 1 shows the characteristics of the study population for the variables of interest. A majority of subjects were women (nearly 80%). Subjects were mostly middle-aged, about half in the age range 40-59 y. More than two thirds of subjects had a university degree and occupations involved mainly sitting or a combination of sitting and standing (for about 75%). More than half of subjects reported that they considered themselves an active person and that physical activity was important or very important for a healthy lifestyle. Half of the sample reported that physical activity and exercise was not a family value. Workday and non-workday sedentary behaviours are shown in Tables 2 and 3 in total and according to socio-demographic characteristics and occupation type, for men and women together.

**Table 1:** **Baseline characteristics of the study population (percentage frequencies (n values))**

***Study population***	***Total***	***N = 35,544***
**Sex, % (n)**	Men	20.8 (7405)
	Women	79.2 (28,139)
**Age, % (n)**	18-39	38.5 (13,688)
	40-59	47.8 (16,989)
	60+	13.7 (4,867)
**Education level, % (n)**	University	71.3 (25,329)
	Secondary	27.2 (9,673)
	Primary	1.5 (542)
**Occupation type, % (n)**	Mainly sitting	42.3 (15,035)
	Combination sitting/standing	32.1 (11,396)
	Mainly standing	12.8 (4,568)
	Some physical effort	10.5 (3,729)
	Heavy manual work	2.3 (816)
**Consider yourself an active person, % (n)**	Yes	58.2 (20,700)
	No	41.8 (14,844)
**Physical activity and exercise was a family value, % (n)**	A lot	14.2 (5,061)
	A little	35.8 (12,718)
	No	50.0 (17,765)
**Importance of physical activity for a healthy lifestyle, % (n)**	Very important	54.7 (19,445)
	Important	39.6 (14,085)
	Less important	5.7 (2,014)

**Table 2:** **WORKDAYS Distribution of sedentary behaviours (hours per day) by sitting and entertainment types on workdays by socio-demographic characteristics and occupation type**

		**WORK SITTING (h/day)**	**TRANSPORT SITTING (h/day)**	**LEISURE SITTING (h/day)**	**TV/DVD TIME (h/day)**	**OTHER SCREEN TIME (h/day)**	**NON-SCREEN TIME (h/day)**
*TOTAL*		*4.17 (3.07)*	*1.10 (1.69)*	*2.19 (1.62)*	*1.53 (1.24)*	*2.19 (2.62)*	*0.97 (1.49)*
**SOCIO-DEMOGRAPHIC CHARACTERISTICS**							
Sex	Men	4.20 (3.11)	1.21 (1.82)	2.29 (1.65)	1.50 (1.24)	2.50 (2.67)	0.99 (1.56)
	Women	4.17 (3.06)	1.08 (1.66)	2.17 (1.60)	1.54 (1.24)	2.11 (2.60)	0.96 (1.47)
		*p:.3451*	***p < .0001***	***p < .0001***	***p:.0347***	***p < .0001***	*p:.2701*
Age	18-39	4.83 (3.00)	1.11 (1.67)	2.22 (1.55)	1.50 (1.21)	2.47 (2.91)	0.94 (1.56)
	40-59	4.21 (3.02)	1.12 (1.72)	2.10 (1.57)	1.51 (1.21)	2.05 (2.52)	0.93 (1.42)
	60+	2.17 (2.58)	1.01 (1.68)	2.42 (1.91)	1.69 (1.40)	1.90 (1.96)	1.21 (1.50)
		***p < .0001***	***p:.0001***	***p < .0001***	***p < .0001***	***p < .0001***	***p < .0001***
Education level*	University	4.54 (3.01)	1.11 (1.66)	2.18 (1.56)	1.43 (1.17)	2.27 (2.71)	0.98 (1.50)
	Secondary	3.32 (3.03)	1.07 (1.75)	2.22 (1.73)	1.77 (1.34)	2.00 (2.37)	0.95 (1.46)
	Primary	2.24 (2.82)	1.29 (2.25)	2.13 (1.75)	2.07 (1.76)	1.93 (2.22)	0.95 (1.34)
		***p < .0001***	***p:.0065***	*p:.1257*	***p < .0001***	***p < .0001***	***p:.0122***
OCCUPATION TYPE**	Mainly sitting	6.21 (2.67)	1.22 (2.02)	2.33 (1.76)	1.52 (1.22)	2.68 (3.13)	1.06 (1.77)
	Combination sitting/standing	3.82 (2.36)	1.10 (1.52)	2.18 (1.53)	1.53 (1.24)	2.16 (2.40)	0.99 (1.38)
	Mainly standing	1.19 (1.43)	0.90 (1.22)	1.97 (1.42)	1.51 (1.24)	1.43 (1.53)	0.81 (1.04)
	Some physical effort	1.45 (1.84)	0.95 (1.26)	2.03 (1.45)	1.57 (1.25)	1.46 (1.61)	0.78 (1.00)
	Heavy manual work	0.71 (1.31)	0.83 (1.11)	1.82 (1.49)	1.67 (1.49)	1.21 (1.34)	0.68 (0.84)
		***p < .0001***	***p < .0001***	***p < .0001***	***p:.0173***	***p < .0001***	***p < .0001***

Data are means (SD).

* Adjusted for sex and age ** Adjusted for sex, age, education level. All p-values are rounded to four decimals.

**Table 3:** **NON-WORKDAYS Distribution of sedentary behaviours (hours per day) by sitting and entertainment types on non-workdays by socio-demographic characteristics and occupation type**

		**TRANSPORT SITTING (h/day)**	**LEISURE SITTING (h/day)**	**TV/DVD TIME (h/day)**	**OTHER SCREEN TIME (h/day)**	**NON-SCREEN TIME (h/day)**
*TOTAL*		*0.85 (1.53)*	*3.19 (2.05)*	*2.24 (1.76)*	*1.85 (1.74)*	*1.30 (1.35)*
**SOCIO-DEMOGRAPHIC CHARACTERISTICS**						
Sex	Men	0.91 (1.59)	3.17 (2.11)	2.09 (1.65)	2.16 (1.86)	1.13 (1.31)
	Women	0.84 (1.51)	3.20 (2.03)	2.28 (1.79)	1.76 (1.69)	1.35 (1.36)
		***p:.0004***	*p:.3734*	***p < .0001***	***p < .0001***	***p < .0001***
Age	18-39	0.83 (1.54)	3.50 (2.22)	2.35 (1.91)	2.15 (2.00)	1.25 (1.42)
	40-59	0.82 (1.45)	2.98 (1.89)	2.16 (1.69)	1.61 (1.53)	1.28 (1.28)
	60+	1.03 (1.73)	3.05 (1.95)	2.20 (1.55)	1.81 (1.47)	1.51 (1.40)
		***p < .0001***	***p < .0001***	***p < .0001***	***p < .0001***	***p < .0001***
Education level*	University	0.78 (1.41)	3.18 (2.01)	2.08 (1.67)	1.80 (1.68)	1.32 (1.34)
	Secondary	1.00 (1.74)	3.22 (2.13)	2.63 (1.89)	1.95 (1.86)	1.26 (1.37)
	Primary	1.45 (2.30)	3.27 (2.44)	3.04 (2.27)	2.12 (2.06)	1.27 (1.54)
		***p < .0001***	***p < .0001***	***p < .0001***	***p < .0001***	***p < .0001***
OCCUPATION TYPE**	Mainly sitting	0.82 (1.60)	3.34 (2.19)	2.22 (1.77)	1.85 (1.82)	1.32 (1.43)
	Combination sitting/standing	0.84 (1.46)	3.11 (1.94)	2.21 (1.71)	1.84 (1.70)	1.33 (1.30)
	Mainly standing	0.90 (1.44)	3.03 (1.88)	2.29 (1.75)	1.88 (1.63)	1.28 (1.30)
	Some physical effort	0.90 (1.48)	3.08 (1.96)	2.34 (1.80)	1.83 (1.66)	1.21 (1.24)
	Heavy manual work	1.05 (1.71)	2.86 (1.92)	2.50 (2.12)	1.80 (1.70)	1.15 (1.22)
		*p:.1443*	***p < .0001***	*p:.5729*	***p:.0185***	***p < .0001***

Data are means (SD).

* Adjusted for sex and age ** Adjusted for sex, age, education level. All p-values are rounded to four decimals.

### Sedentary behaviour on workdays

Mean time spent sitting was 4.17 h/d at work, 1.10 h/d in transport, and 2.19 h/d in leisure on workdays. The most time-consuming sedentary entertainment was “other screen activities” with an average 2.19 h/d spent in these pursuits, followed by 1.53 h/d spent on TV/DVD viewing, and 0.97 h/d in non-screen activities.

### Sedentary behaviour on non-workdays

Mean time spent sitting was 0.85 h/d in transport and 3.19 h/d in leisure on non-workdays. TV/DVD viewing was the principal sedentary entertainment with 2.24 h/d, followed by 1.85 h/d spent in other screen activities, and 1.30 h/d in non-screen activities.

### Associations with gender, age and education

On workdays (Table 2), men reported significantly more time spent in all sedentary behaviours, except for work sitting (ns), and for TV/DVD viewing time, which was higher in women. On non-workdays (Table 3), the gender differences were less consistent across sitting time domains and sedentary leisure activities. As for age, young adults (18-39 y) reported in general the highest amount of sitting and entertainment time, irrespective of day type. The contribution of work sitting to total sitting was largest in young adults, representing 59% of the total sitting time. For older adults (≥60 y) this contribution was 39%. As regards sedentary entertainment, the age trend was opposite in TV/DVD time for workdays and non-workdays, with the highest time spent in TV/DVD viewing in older adults on workdays, and in young adults on non-workdays. Non-screen time was higher in older adults, and other screen time was consistently higher in younger adults for both workdays and non-workdays.

For education, there was an overall gradient of increased sitting time with increased education level on workdays (Table 2). Time spent in other screen and non-screen behaviours also increased with increasing education level, while TV/DVD decreased with increasing education level, for workdays. For non-workdays (Table 3), all sitting domains, TV/DVD time and other screen time decreased with increasing education level. Only non-screen time increased with increasing education level.

### Associations with occupation type

A substantial proportion of subjects reported their occupation involved mainly sitting (42.3%) (Table 1). For workdays, a mainly sitting occupation was significantly associated with increased time spent in all sitting domains (including work) and all sedentary entertainments (Table 2). The only exception was time spent viewing TV/DVD, which by large followed an opposite positive trend: the more strenuous the occupation, the more time spent viewing TV/DVD on workdays. For non-workdays (Table 3), patterns of associations were less clear, but a mainly sitting occupation was associated with more sitting during leisure, and more screen time other than TV/DVD and more non-screen time.

### Associations with perceptions towards physical activity

**Figure 1:**
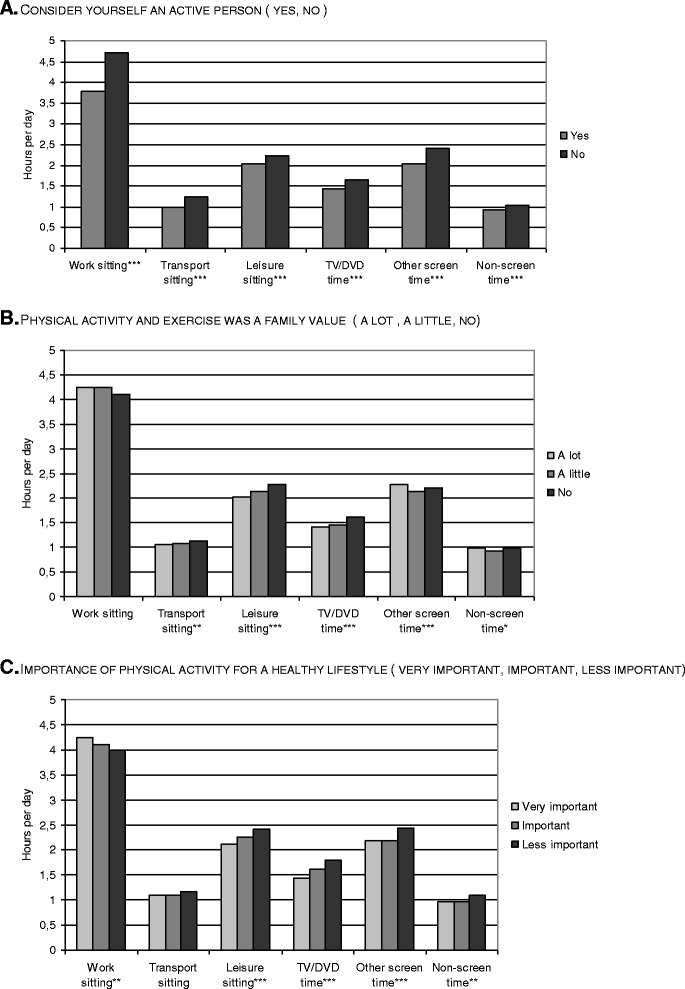
Workdays sedentary behaviours and physical activity perceptions. **A.** Consider yourself an active person (yes, no). **B.** Physical activity and exercise was a family value (a lot, a little, no). **C.** Importance of physical activity for a healthy lifestyle (very important, important, less important).

**Figure 2:**
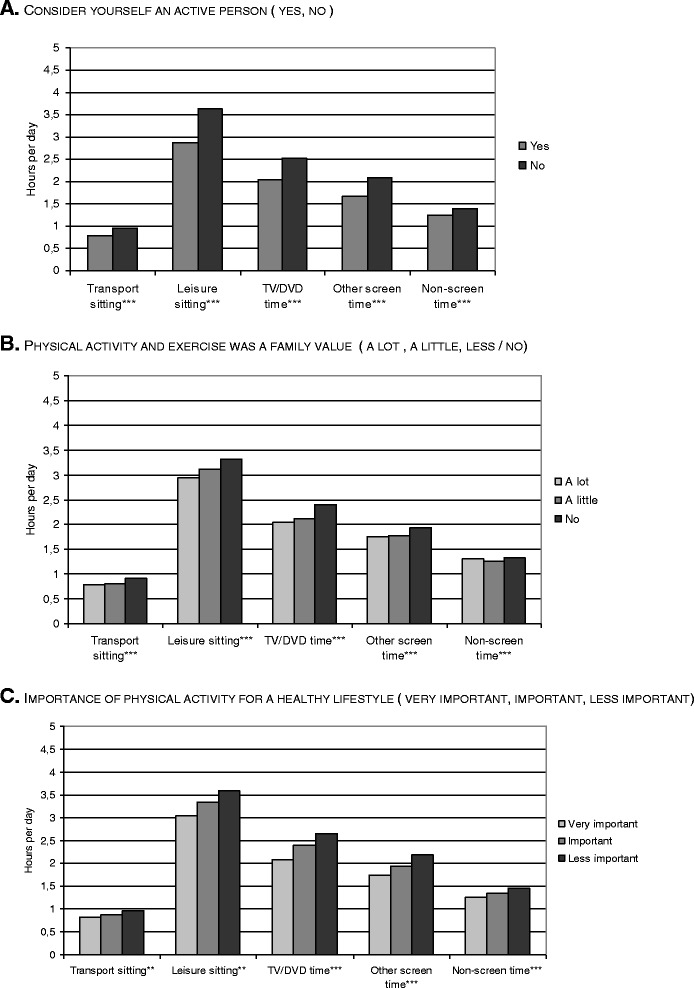
Non-workdays sedentary behaviours and physical activity perceptions. **A.** Consider yourself an active person (yes, no). **B.** Physical activity and exercise was a family value (a lot, a little, less / no). **C.** Importance of physical activity for a healthy lifestyle (very important, important, less important).

Associations with perceptions towards physical activity are presented graphically for workdays (Figure 1 A-C) and non-workdays (Figure 2 A-C). There was a statistically significant negative association between *perceiving oneself as an active person* and time spent in all sitting domains and entertainments on workdays (1.A). Likewise, *having grown up in a family attaching value to physical activity and exercise* was significantly negatively associated with time spent in transport sitting, leisure sitting, and TV/DVD viewing on workdays (1.B). Likewise, *perceiving physical activity as important for a healthy lifestyle* was significantly negatively associated with time spent in leisure sitting and all sedentary entertainments on workdays (1.C), but the association with work sitting was inverse, i.e. the more important physical activity was perceived, the higher the time sitting at work (1.C). For non-workdays, significantly negative associations were consistent for all sitting and entertainment time, across all three types of perception towards physical activity (2.A-C), i.e. the more negative the perceptions were, the more sedentary behaviour.

## Discussion

The objective of this study was to explore a wide range of self-reported sedentary behaviours in terms of amount and context, by day type, occupation type, and perceptions towards physical activity in a large sample of French working adults. As expected and in line with other studies [14,18,28], work sitting accounted for the majority of sedentary behaviour on workdays. TV/DVD viewing was the main sedentary entertainment on non-workdays, whereas computer-based activities including tablets and playing inactive video games were the main sedentary entertainment on workdays. Interestingly, this was observed in all age groups, even in subjects aged ≥ 60 years. This may question the use of TV time as indicator for total sedentary leisure-time [2,21,29], as it may no longer be the most common sedentary behaviour that adults engage in. We found that different sedentary behaviours may have similar socio-demographic correlates, but in opposite directions, in line with previous research [5]. The findings differed for workdays and non-workdays, which suggest that the design of future interventions may need to take into account the differences in behaviour between working and non-working days. Of particular interest we found that workers with mainly sedentary jobs reported the highest sitting time outside of work on both workdays and non-workdays, and reported the highest sedentary entertainment time (on workdays). This could suggest that being sedentary at work is not compensated with less sedentary behaviour outside of work; rather there could be an accumulating sedentary effect of a sedentary occupation. This finding is supported by the few other studies with a similar focus [14-16], reporting that workers with jobs involving mostly sitting are more likely than workers with mostly heavy labour jobs to have high leisure-time sitting; also supported by objective monitoring of sedentary behaviour using accelerometry [15,16].

In an attempt to uncover the socio-ecologic breadth of sedentary behaviours and its behavioural determinants [6], we explored the association with perceptions towards physical activity. We found in general that workers with negative perceptions towards physical activity spent more time in sedentary behaviours on both workdays and non-workdays. The association was, however, less consistent for work sitting, suggesting differences in correlates at work and outside work, and/or residual socio-economic confounding, not captured by our adjustment for education level. Few studies have reported on perceptions in relation to sedentary behaviours: a study conducted in youth reported that sedentary behaviour was negatively associated with selected physical self-perceptions irrespective of physical activity [30], and two studies in adults found that perceived barriers to physical activity (cost, weather) were also correlates of TV viewing [31,32]. The importance of addressing one’s attitudes and beliefs is well recognized in order to maximize the effectiveness of exercise programs [33,34]. It is therefore interesting for future studies to look more closely at the perception aspect to improve our understanding of sedentary behavioural choices. Perceptions are considered to be a component of one’s self-concept, which culminate as a directing force in behaviour, and as such individual perceptions are likely to trigger/contribute to the amount of sedentary behaviours, and perceptions (positive/negative) towards physical activity may influence ones choice of sedentary behaviours.

### Strengths and limitations

The specific strengths of this study include the large sample size allowing for stratified analyses, and the fact that sedentary behaviours were assessed both for workdays and non-workdays, in continuous form, in a wide range of domains (work, transport, leisure) by both sitting time and specific types of sedentary entertainment. A potential limitation relates to concurrent behaviour: as the nature of sedentary behaviour often involves doing multiple things at the same time, e.g., using a tablet while viewing TV, and/or viewing TV on your tablet, there is a risk of duplicate reporting. Duplication of time may explain the dissimilarity with the amounts reported for France in the Eurobarometer study 2012 where 44% of adults report sitting 2h31min to 5h30min, and only 26% report sitting 5h31min to 8h30min [35], yet it was just assessed with a single sitting question, irrespective of sitting domain or day type.

Questionnaire limitations must be noted. Estimates of time are subject to recall errors, social desirability bias and difficulties with correctly capturing the amount of individual sedentary behaviour. Since sedentary behaviours occur in a sporadic manner throughout the day, self-reporting sedentary behaviour is complicated. Employing one or few sedentary behaviours as an overall marker of sedentary behaviour can result in underestimation of total sedentary behaviour, as it does not include specific types of sedentary behaviours [1,36]. We have addressed a wide range of sedentary behaviours, assessed by two items (sitting time and sedentary entertainment time), each capturing different types and domains of sedentary behaviours. It is recognized that self-reported and objective sedentary behaviour each provides distinct information, but are complementary. Objective monitoring is important for providing data on activity patterns, while subjective measures remains important because they provide domain-specific information [3,21]. Psychometric properties of the sedentary behaviour questionnaire used in this study are comparable to those of other instruments that have been validated in adults [21].

We must also draw attention to the validity of the physical activity perception questions, which have not been assessed with standardized instruments, but are similar to the ones included in validated and commonly used SDT-based instruments (e.g., the Intrinsic Motivation Inventory, [27]).

Our sample included proportionally more women and more individuals of high educational levels as observed in volunteer-based studies [37]. As imposed by the study objective, the study population consists of those in employment (counting those in paid/unpaid employment and those studying). A “healthy worker effect” might be present (the severely ill and chronically disabled are ordinarily excluded from employment), and our findings are thus representative of a healthy and resourceful population of adults. Furthermore, 90% of our study participants live in an urban area. Socio-cultural attributes do also impact the generalizability of our findings. Bauman et al. [36] showed differences in prevalence and gender variations in adults’ overall sitting time across the 20 countries examined in their study, and thus population generalization should be made cautiously. Lastly, a study limitation is the cross-sectional design, not allowing causal interpretations of the results. Hence we cannot preclude that the findings may be an expression of sedentary workers who self-selected a sedentary occupation and have some predisposition to be sedentary.

## Conclusions

This explorative study takes a step in providing insight into the understanding of sedentary behaviours and the context in which these behaviours take place. The findings demonstrate that sedentary behaviour is multi-faceted and require more detailed assessment than can be obtained by markers of overall sitting time. The relationship is complex as each type of sedentary behaviour is differently associated with socio-economic and occupational status, on working and non-working days.

Moving populations from sedentary behaviours towards more light intensity activity might be a more realistic approach with substantial effects on public health, rather than solely focusing on increasing physical activity of moderate-to-vigorous intensity. Based on our results, we propose to address the accumulation of sedentary behaviour outside work for those with sedentary occupations, and advise awareness of the possible impact of perceptions of physical activity on ones accumulation of sedentary behaviours.
